# Discovering tungsten-based composites as plasma facing materials for future high-duty cycle nuclear fusion reactors

**DOI:** 10.1038/s41598-024-64614-3

**Published:** 2024-06-15

**Authors:** Trevor Marchhart, Chase Hargrove, Alexandru Marin, Hanna Schamis, Ashrakat Saefan, Eric Lang, Xing Wang, Jean Paul Allain

**Affiliations:** 1 Ken and Mary Alice Department of Nuclear Engineering, Pennsylvania State University, University Park, PA 16801 USA; 2 Surface Analysis Laboratory, Institute for Nuclear Research Pitesti, 115400 Mioveni, Romania; 3 Department of Nuclear, Plasma and Radiological Engineering, University of Illinois at Urbana-Champaign, Urbana, IL 61801 USA; 4 Department of Nuclear Engineering, University of New Mexico, Albuquerque, NM 87106 USA

**Keywords:** Nuclear fusion and fission, Structural materials

## Abstract

Despite of excellent thermal properties and high sputtering resistance, pure tungsten cannot fully satisfy the requirements for plasma facing materials in future high-duty cycle nuclear fusion reactions due to the coupled extreme environments, including the high thermal loads, plasma exposure, and radiation damage. Here, we demonstrated that tungsten-based composite materials fabricated using spark-plasma sintering (SPS) present promising solutions to these challenges. Through the examination of two model systems, i.e., tungsten-zirconium composite for producing porous tungsten near the surface and dispersoid-strengthened tungsten, we discussed both the strengths and limitations of the SPS-fabricated materials. Our findings point towards the need for future studies aimed at optimizing the SPS process to achieve desired microstructures and effective control of oxygen impurities in the tungsten-based composite materials.

## Introduction

The discovery of new materials to withstand the combination of particle and heat fluxes under the extreme conditions expected in high-duty cycle nuclear fusion reactors remains one of the most daunting challenges to realizing the ability to provide firm, reliable, and sustainable energy from nuclear fusion reactions. Early fusion experimental reactors transitioned from stainless steel plasma-facing materials (PFMs) to low-Z based materials, namely graphite, due to radiation losses at the edge of fusion plasma bounded by the reactor wall^1^. Further designs included the development of limiter and divertor systems that enabled early experimental tokamak fusion reactors to reach higher performance operation. Notably JET and TFTR were fusion machines that achieved breakeven conditions by their use of graphite-based PFMs. Subsequent development included the realization of wall conditioning and in TFTR the use of lithium-based coatings to improve confinement linked to its impurity-gettering properties and low-Z favorable conditions minimizing radiation losses. However, graphite-based PFMs, although provided a pathway to enhanced confinement, were compromised by chemical sputtering effects with temperature sensitive regimes that did not allow for enhanced fusion power operation at longer duty cycles due their entrainment of hydrogen and transport of T fuel to vacuum ducts, rendering this approach an unknown safety risk. The transition to higher Z PFMs was premised by the fact that low H retention was expected in these materials and low sputtering from fusion reaction light particles including hydrogen isotopes and low-energy He particles transported from the core to the edge^2^. However, this transition also had to contend with plasma-material interaction (PMI) regimes that needed favorable properties to high-duty cycle operation of a fusion reactor. These included tolerating temperature-dependent variation of mechanical properties of the material and synergistic effects of neutron-induced mechanisms on PMI behavior.

The balance of addressing both PMI properties and thermo-mechanical properties of the structural armor material is one of the emerging areas of PFM design for future high-duty cycle PFMs. This balance is focused on devising materials that can go beyond the limit of conventional high-temperature materials to significant thermo-mechanical property losses due to degradation from neutron-induced effects and plasma-driven chemical interactions at the PMI under high-duty cycle effects in tokamak-based fusion reactor environments. For example, in future Fusion Pilot Plants (FPP), the PFMs must withstand various degradation mechanisms caused by high-duty cycle operations, including > 100-dpa radiation damage, > 1000-appm He production, extremely high steady-state heat loads (~ 10 MW/m^2^), and transient thermal shocks (~ GW/m^2^)^3,4^. It can be a daunting task for single-component or monolithic materials to meet all the requirements. For instance, tungsten (W) is a leading candidate as PFMs because of its high melting point, excellent sputtering resistance, and low hydrogen retention. However, embrittlement of W due to high-temperature recrystallization and radiation damage significantly limit its operational window. Conversely, W-based composite materials present a unique opportunity to address these challenges^5^. By integrating the strengths of each component, the composites have the potential to provide multi-functionality and improve resistance to the extreme environments for FPP PFMs. One good example is dispersion-strengthened tungsten (DSW), where the grain growth of the W matrix is suppressed by intergranular oxide or carbide dispersoids, thus achieving refined grains and higher resistance to recrystallization^6,7^. Another good example is the liquid metal PFM supported by a porous W substrate. This two-phase composite or hybrid material allows the liquid metal to self-heal from radiation damage while the porous W substrate stabilizes the thin metal liquid layer facing the fusion plasma^8–10^.

Spark plasma sintering (SPS), including placeholder spark plasma sintering (PHSPS), has been proven as an optimal method for fabricating refractory alloys and composites because of its capability to produce high-density materials at lower sintering temperatures and shorter sinter time, thus offering better microstructure control than conventional powder metallurgy methods^11,12^. SPS has been extensively employed to create various DSW embedded with carbide and oxide particles at micro- or even nano-meter sizes^11,13^. In PHSPS, a precursor containing both the desired component and less noble components is fabricated by SPS first. The less noble components are subsequently removed by dissolution or sublimation, yielding porous materials^14^. PHSPS has been successfully applied for producing porous Ti_6_Al_4_V foams with NaCl as the placeholder and W_6_Fe_6_C-lined porous W structures with Fe as the placeholder, achieving a high porosity of around 70% in both final products^14,15^.

Despite these promising advancements, significant knowledge gaps remain in elevating SPS-derived W composites to the next technology readiness level as FPP PFM. Notably, more precise control over the composite microstructures, including the phases formed, grain sizes, and impurities, is needed. Furthermore, it is crucial to evaluate the performance of these composites under fusion-reactor-relevant conditions. Most importantly, comprehensive studies are required to clarify the fundamental link between the resulting microstructures and their performance. Understanding this relationship is critical for further refining the SPS process, ultimately enhancing the performance of these materials in fusion reactors.

In this paper, we spotlighted our recent efforts to tackle the aforementioned challenges in two model systems: (i) the W-Zr precursor for fabricating porous W, and (ii) DSW doped with ZrC dispersoids. Specifically, we employed the W-Zr system to illustrate how sintering parameters, such as temperature and pressure, can be fine-tuned to control the ratio of desired phases and enhance the manufacturability of porous W. We also examined the surface stability of W-ZrC under high heat fluxes both at steady and transient tokamak operation conditions, revealing the key roles of microstructures on material performance in fusion reactor-relevant conditions. In both the W-Zr and W-ZrC systems, oxygen appears to be a common major challenge that complicates the functionality of W composite materials. The origin, impact, and potential controlling strategies of these oxygen impurities will be discussed.

## Results

### Impact of sintering process on composite microstructures

#### Effects of temperature and pressure on phase distribution in W-Zr precursor for porous W

**Figure 1:**
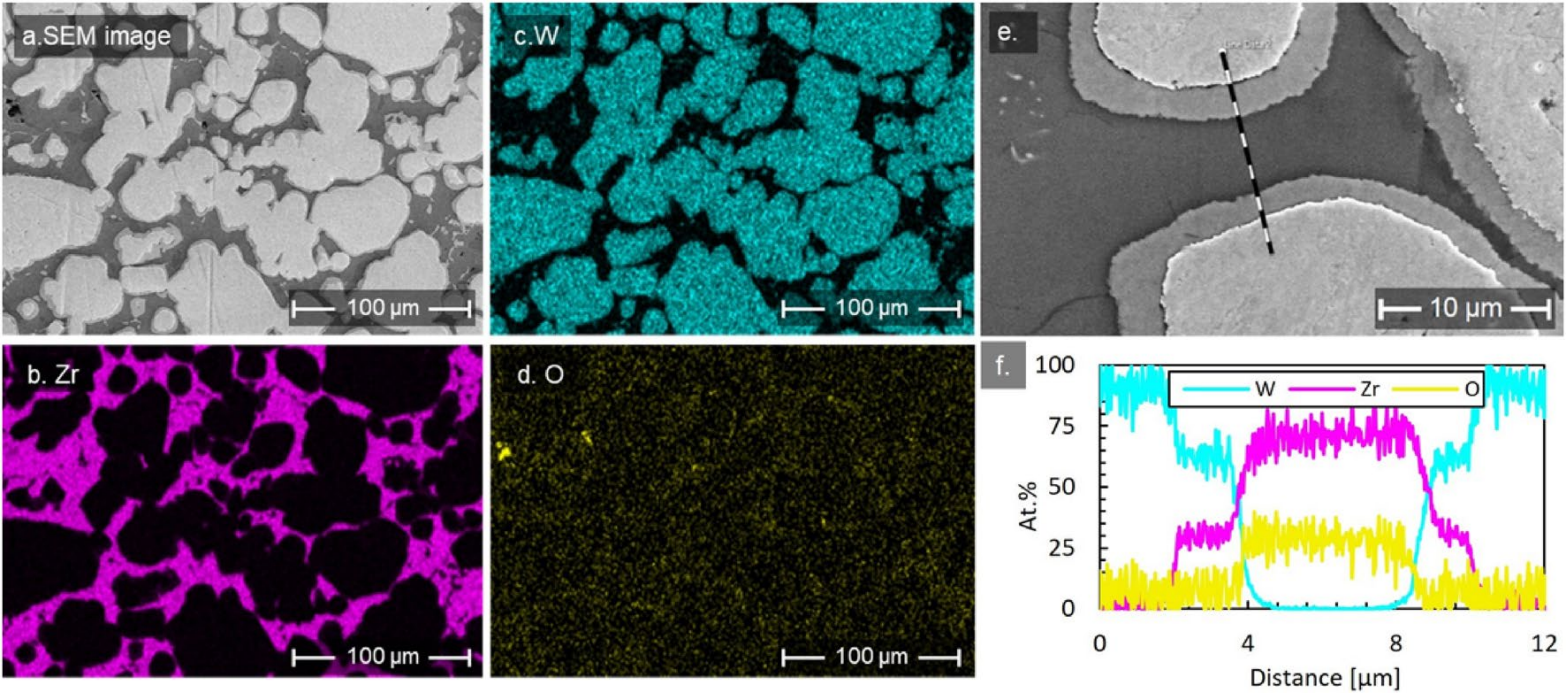
Microstructure and element distribution in W-Zr Sample 1. (**a**) SEM image; (**b**) EDS mapping of Zr, (**c**) W, and (**d**) O; (**e**) SEM image showing the position of EDS line scan; (**f**) Element composition profiles along the line.

PHSPS has been applied to fabricate the W-Zr precursors, in which the Zr will be dissolved in acids for producing porous W. Zr was ultimately selected as a placeholder due to its relatively high melting point of 1855 °C, its ability to easily dissolve in nitric and sulfuric acid mixtures^16^, and its corrosion resistance to molten lithium^17^. Practically, it is difficult to ensure that 100% of the precursor material is dissolved out of the sample, so compatibility with the liquid metal is critical. These properties allow for fabrication with tungsten in SPS processes and ensure chemical compatibility of all constituents with the desired liquid metal integration. The general properties of W-Zr precursors are well displayed by the sample sintered at 1700 °C with applied pressure at 50 MPa. For simplicity, this sample is named Sample 1 throughout the rest of the paper. As shown in Fig. 1a–d, the scanning electron microscopy (SEM) image and energy dispersive X-ray spectroscopy (EDS) analysis revealed that W particles (roughly ~ 20–50 µm in size) are separated by the Zr matrix. Besides two principal elements, impurities including oxygen and carbon were also found by EDS element mapping. The uniformly distributed carbon is likely to be surface contamination, but oxygen appears to be spatially correlated with Zr (Fig. 1d). To obtain a more quantitative characterization of element distributions, an EDS line scan was conducted across two W particles. As shown in Fig. 1e–f, W particles are surrounded by a mixed layer with constant compositions of about 65 at.% W and 35 at.% Zr. According to the W-Zr phase diagram^18^, it is likely that the intermixing layer is the intermetallic W_2_Zr phase. The line scan also confirms that oxygen is mostly concentrated in the Zr matrix. Considering EDS is not the most suitable tool for characterizing light elements such as carbon and oxygen, impurities will be systematically analyzed using X-ray photoelectron spectroscopy (XPS) in the next section.

**Figure 2:**
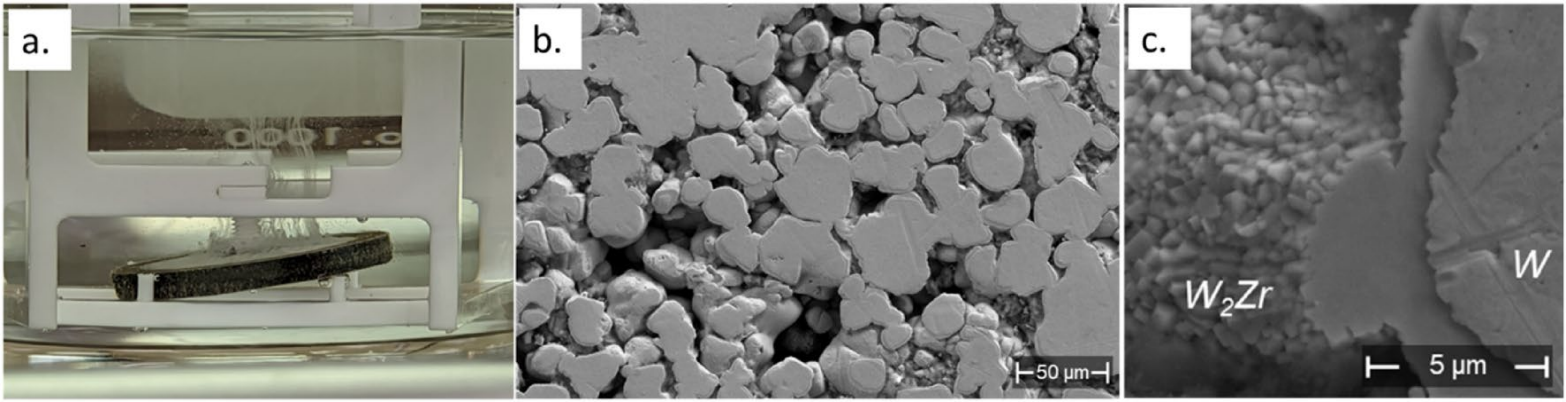
Production and examination of porous W on the surface of Sample 1. (**a**) Acid corrosion process; (**b**) SEM image showing the surface of porous W; (**c**) Patterned surface of W_2_Zr.

The Sample 1 precursor was then submerged in a 1HNO_3_: 9H_2_SO_4_ acid solution to dissolve the Zr portions. As shown in Fig. 2a, captured 30 min after the precursor was placed in the acid, visible particulates came from the precursor surface, indicating the chemical reaction was taking place. After about 25 h, no more fluxes of particulates could be observed. The precursor sample was taken out of the acid and cleaned. SEM image in Fig. 2b confirmed that most Zr near the surface has been etched away and porous W is produced on the surface. As detailed in Supplementary Materials, cleavage and imaging of the cross section revealed that only the top ~ 150 µm of Zr was dissolved. Although the goal of such samples is to fully dissolve the precursor, full dissolution is not entirely critical given this specific application. Dissolution of just the surface of the placeholder material will still allow for containment of the liquid metal^8–10^. Replenishment of the liquid metal can then be achieved through other means such as machined channels. Additionally, it is anticipated that by optimizing the chemical reaction procedures, such as employing larger acid volumes and raising the acid solution temperature, more accessible Zr could be effectively dissolved from the precursor. Closer inspection of dissolved samples found that the intermetallic W_2_Zr phase remains largely intact, leaving behind a rougher surface with a patterned structure (Fig. 2c). It is expected that the rough surface of the intermetallic phase could augment the wettability of liquid metals, such as lithium, thus facilitating the refilling process of liquid lithium in the porous wall^8^. Meanwhile, since the W_2_Zr phase is insoluble in the acid, it would inhibit the further dissolution of Zr and influence the production of porous W. Therefore, additional samples were fabricated with varying sintering parameters to better understand the formation of the W_2_Zr intermetallic.

**Figure 3:**
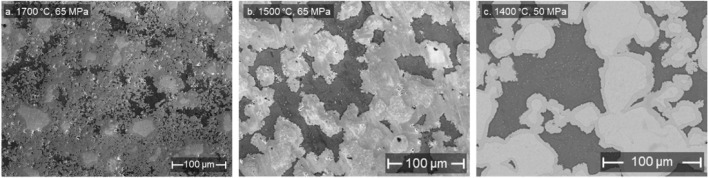
Impact of sintering temperature and hold pressure on the formation of W_2_Zr phase. SEM image of (**a**) Sample 2 precursor; (**b**) Sample 3 precursor; and (**c**) Sample 4 precursor. In the SEM images, high-, median-, and low-intensity regions are W, W_2_Zr, and Zr, respectively.

Sample 2 precursor was fabricated with higher sintering parameters than Sample 1, at 65 MPa and 1700 °C. Sample 3 precursor was sintered at slightly reduced conditions of 65 MPa and 1500 °C, and Sample 4 precursor had the lowest at 50 MPa and 1400 °C. The resulting microstructures of precursors for Sample 2, 3, and 4 are shown in Fig. 3a, b, and c, respectively. Sample 2 precursor displays significant formation of W_2_Zr intermetallic extending far past just the borders of the W agglomerations. This effect would limit the amount of Zr that is exposed to the surface and can be dissolved by the acid mixture, making it more difficult to create a porous surface. As the sintering conditions become less intense with Samples 3 and 4, the formation of the intermetallic extended less far from the W surface, which is similar to Sample 1. Thus, lowering the sintering temperature and pressure is helpful to control the formation of the W_2_Zr intermetallic. This trend can be attributed to the fact that local melting of Zr could occur above 1650 °C during the SPS process^19^. It is hypothesized that the presence of liquid Zr enhances the transport and formation of W_2_Zr away from the larger W groupings. This comparison is harder to make against Sample 1, as a smaller powder size and larger W/Zr ratio was used for that sample (see Table 3). To obtain preliminary measurements of the mechanical properties of the W-Zr composite with a porous tungsten surface, we conducted miniature three-point bending tests on Samples 1 and 4. As detailed in the Supplementary Materials, these samples exhibit strength and ductility comparable to bulk W and Zr, suggesting the potential of these composites as future PFCs.

### XPS characterization of impurities in porous W

**Figure 4:**
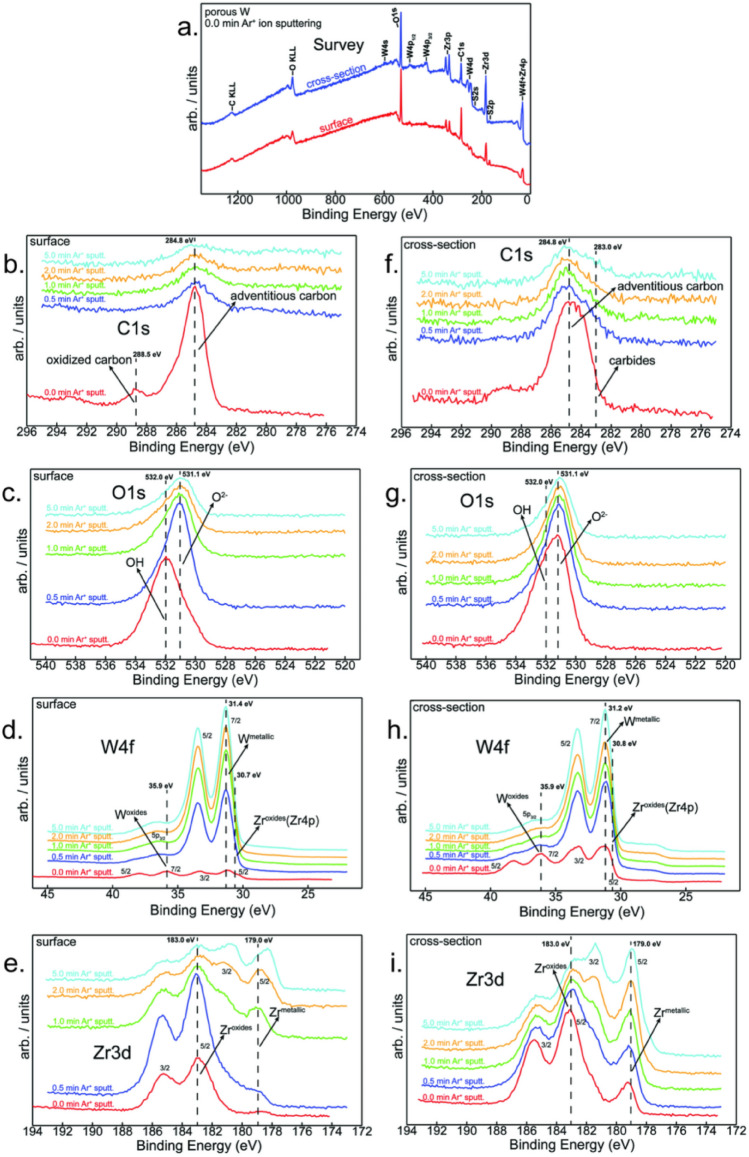
XPS analysis of porous W surface and cross-section of Sample 3. (**a**) Superimposed general XPS spectra acquired from the surface (in red) and cross-section (in blue) of porous W; (**b**–**e**) Etching time-dependent XPS peaks of C, O, W, and Zr on analyzed surface, respectively; (**f**–**i**) Etching time-dependent XPS peaks of C, O, W, and Zr on analyzed cross section, respectively.

Although porous W was created near the surface of the W-Zr precursor, cross-sectional images showed that the porous layer is only about 150 µm thick, even after the precursor had been immersed in acid for 25 h. Since EDS analysis detected a high concentration of oxygen in correlation with the Zr matrix, it suggests that Zr oxide may form within the Zr matrix, thus potentially hindering the dissolution of Zr in acids. Therefore, it is necessary to fully understand the distribution and chemical status of oxygen. To achieve this goal, XPS analyses were conducted on the porous W surface of Sample 3, as well as its newly cleaved cross section. Since the cross-section had not been in contact with the acids, it represents the bulk chemistry of the precursor. Consistent with EDS results, the wide-scan spectra of both the surface and cross-section in Fig. 4a have clear C and O peaks, demonstrating the presence of these impurities. To characterize the element distributions along the depth profile, Ar^+^ ion sputtering was applied to the specimens with a fixed amount of time and then the depth-dependent XPS signals were acquired. On average, about 1.7 nm of materials was removed per minute of sputtering. Narrow region scans for C, O, W, and Zr are plotted in Fig. 4b–e for the porous W surface, and in Fig. 4f–i for the cross-section. The binding energies associated with certain chemical statuses of each element are marked as black dashed lines in these figures. For more quantitative comparisons, the depth-dependent element concentrations calculated from XPS results are also summarized in Table 1 for the surface and in Table 2 for the cross section. As shown in Fig. 4b and f, the C peaks drop quickly with sputtering time on both specimens, suggesting that the C contamination is specific to unavoidable surface contaminants during the sample handling process, e.g., hydrocarbon from the ambient environment. Additional C that remains in the sample is likely due to contamination from the graphite tooling (dies, punches, protective foil) during sintering. In contrast, strong oxygen peaks persist with Ar^+^ sputtering and similar oxygen concentrations of about 26–27 at.% are found in the surface and cross-section specimens after 5-min sputtering, indicating that the oxygen impurity exists in the bulk of the W-Zr precursor and may be introduced during the sintering process. It is worth noting that the surface specimen contains more hydroxyl (OH) groups located at 532.0 ± 0.2 eV than the cross-section specimen^20,21^, which is consistent with the fact that the surface was exposed to acid solutions, potentially leaving behind residual moistures.

**Table 1:** Element relative concentrations (at.%) on the surface of porous W.

Surface element composition	C1s	O1s	Zr3d	W4f
0.0 min Ar^+^ ion sputtering	55.0	36.8	4.8	3.4
0.5 min Ar^+^ ion sputtering	19.4	43.7	14.8	22.1
1.0 min Ar^+^ ion sputtering	15.9	35.3	12.3	36.5
2.0 min Ar^+^ ion sputtering	11.6	30.0	11.9	46.5
5.0 min Ar^+^ ion sputtering	6.8	26.1	12.2	54.9

**Table 2:** Element relative concentrations (at.%) on the cross-section of W-Zr precursor.

Cross element composition	C1s	O1s	Zr3d	W4f
0.0 min Ar^+^ ion sputtering	40.3	40.3	10.9	8.5
0.5 min Ar^+^ ion sputtering	27.3	38.1	17.6	17.0
1.0 min Ar^+^ ion sputtering	22.4	36.1	20.2	21.3
2.0 min Ar^+^ ion sputtering	20.0	32.9	22.4	24.7
5.0 min Ar^+^ ion sputtering	16.7	27.2	25.4	30.7

W and Zr also behave differently. Initially, a W oxide peak was observed at 35.9 eV, but W swiftly transitions to a pure metallic state after just 5 min of Ar^+^ sputtering in both surface and cross-section specimens, indicating that W oxidation only occurred within the initial few nanometers of the surface, likely induced by sample exposure to air. Contrastingly, after the same 5-min Ar^+^ sputtering, distinct peaks indicative of a fully oxidized Zr state at 183.0 ± 0.2 eV^22^ and a metallic Zr state at 179.0 ± 0.2 eV^22,23^ are clearly visible in both the surface and cross-section specimens. This suggests the presence of a mixture of metallic and oxidized Zr beneath the specimen surface. These contrasting chemical states between Zr and W imply that Zr functions as an oxygen getter, protecting W from further oxidation and reducing its higher valence state. This finding aligns with the strong chemical affinity of Zr for oxygen^24,25^, and the source of this oxygen can be traced back to the fact that SPS was performed in only low vacuum (~ 5 Pa) for these samples. More careful examinations of the surface specimen found that the intensity of the metallic Zr peak at 179 eV increases with increasing sputtering time. Accordingly, the Zr concentration in Table 1 rises from 4.8 at.% before sputtering to 12.2 at.% after the Ar^+^ sputtering. Even though the surface has been subjected to corrosion in acid solutions, a distinct metallic Zr peak is detectable beneath the surface, suggesting that the Zr oxide layer could shield the underlying Zr metal from acid dissolution.

### Microstructure and chemistry of DSW fabricated by SPS

**Figure 5:**
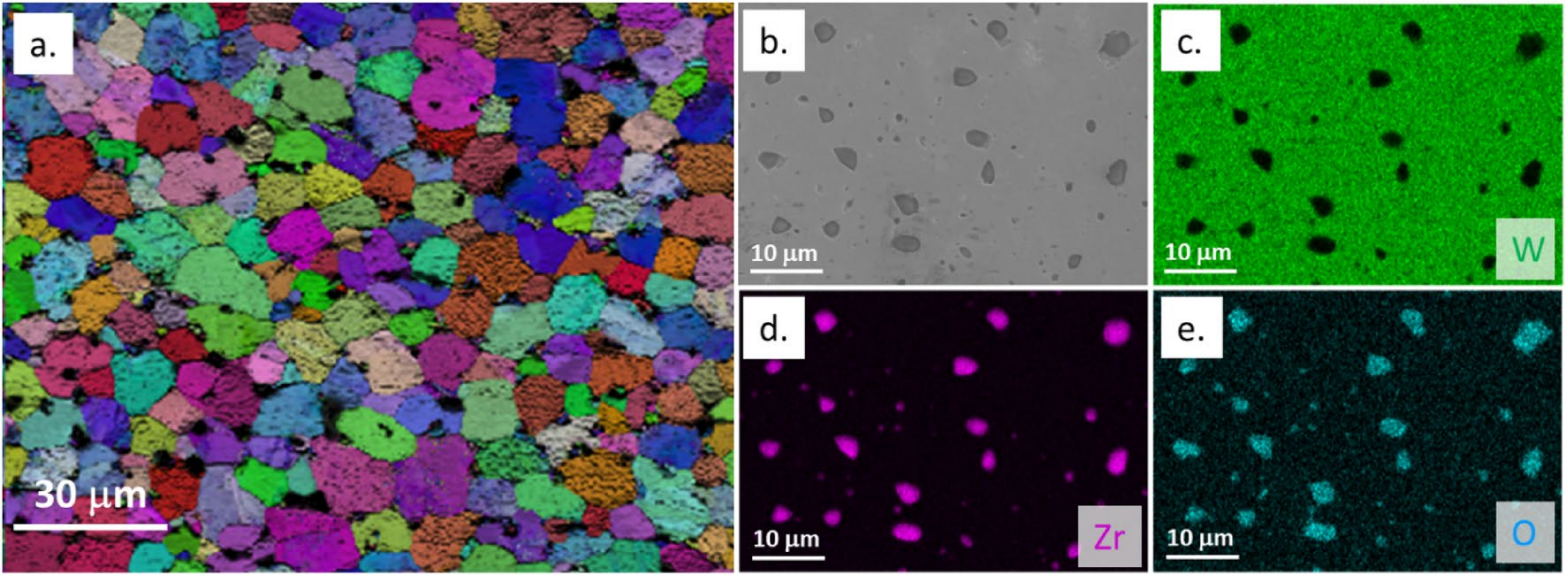
Microstructure of W-1.1ZrC. (**a**) EBSD maps showing most dispersoids embedded between W grains, where W grains are colored depending on their crystal orientation and dispersoids are black; (**b**) SEM image showing the size of dispersoids; (**c**–**e**) EDS element mapping of W, Zr, and O, respectively. Figure 5a is reprinted from E. Lang, et al., *Recrystallization Suppression Through Dispersion Strengthening of Tungsten*, Journal of Nuclear Materials, 545, (2021) 152631, with permission from Elsevier.

In our previous studies, SPS had been employed to fabricate DSW materials doped with three different types of carbide dispersoids, i.e., ZrC, TiC, and TaC, at concentrations ranging from 1 to 10 wt.%^6^. The average sizes of dispersoid particles and W grains depend on the type and concentration of the dispersoids. However, in general, all DSW materials displayed similar microstructure with micro-sized dispersoids uniformly distributed between W grains. A typical example of W-1.1 wt.%ZrC was illustrated in Fig. 5a. For ease of reference, we will refer to this sample as W-1.1ZrC throughout the paper and, similar simplification will be applied to all the DSW samples. Studies have demonstrated that these second-phase dispersoids can effectively improve the mechanical properties of pure W. Notably, the intergranular dispersoids can pin grain boundary motion, thus suppressing recrystallization and improving the material strength at elevated temperatures^26^. Furthermore, as the Group IVB and VB transition metal carbides have a strong chemical affinity to oxygen, they would help attract oxygen impurities that typically concentrate near grain boundaries in the W matrix, consequently enhancing the material ductility^2^. Our EDS analysis did not find evidence for the formation of detrimental tungsten oxides or tungsten carbides, which is consistent with our previous findings^﻿27^.

Like the W-Zr precursors, knowledge gaps exist for understanding and controlling the distribution of oxygen impurity in the DSW system. As shown in Fig. 5b–e, EDS element mapping of as-fabricated W-1.1ZrC indicates there is a strong spatial correlation of oxygen and Zr, suggesting that the carbide particles have been transformed into a carbide-oxide mixture, which was also reported in the literature^14^. This phenomenon aligns very well with the thermodynamics calculation showing that oxides of Group IVB and VB metals (e.g., ZrO_2_, TiO, Ta_2_O_5_) have much lower Gibbs formation energy than Group VIB metal oxides (e.g., W and Hf oxides)^28^. Studies also reported that ZrC acted as an efficient oxygen getter^29^. Therefore, the dispersoids are more attractive to oxygen than the W matrix. From the perspective of material performance, on one side, the accumulation of oxide near dispersoids helps purify the W matrix and improve the ductility; on the other side, the oxidation of carbides can significantly modify the thermomechanical properties of carbide dispersoids. For instance, the thermal conductivity of ZrO_2_ is an order of magnitude smaller than ZrC^29,30^. Therefore, further investigations about the impact of oxygen impurity on plasma-material-interaction properties of DSW are still needed.

## Performance evaluation under fusion reactor-relevant conditions

In a fusion reactor environment, divertor PFMs will be subjected to both extreme heat and particle loads^31^. In addition to normal operation temperatures around 800–1000 °C, divertor PFMs also face steady state heat loads on the order of 10–20 MW/m^2^, while transient events such as edge-localized modes (ELMs) and vertical displacement events (VDEs) can deposit several GW/m^2^ over the course of 0.5–1 ms^32^. These transient heat loads can induce thermal shock events such as localized recrystallization and cracking onto the surface of divertor PFMs. Further challenges arise with the implantation of hydrogen isotopes into the PFM surface. This process can cause severe degradation, such as embrittlement, blistering, and cracking, thereby reducing the material resistance to thermal shocks^33^. Hence, it is vital to study both the individual and combined effects of high heat flux and particle flux.

### Evolution of surface morphology of DSW under high heat flux

**Figure 6:**
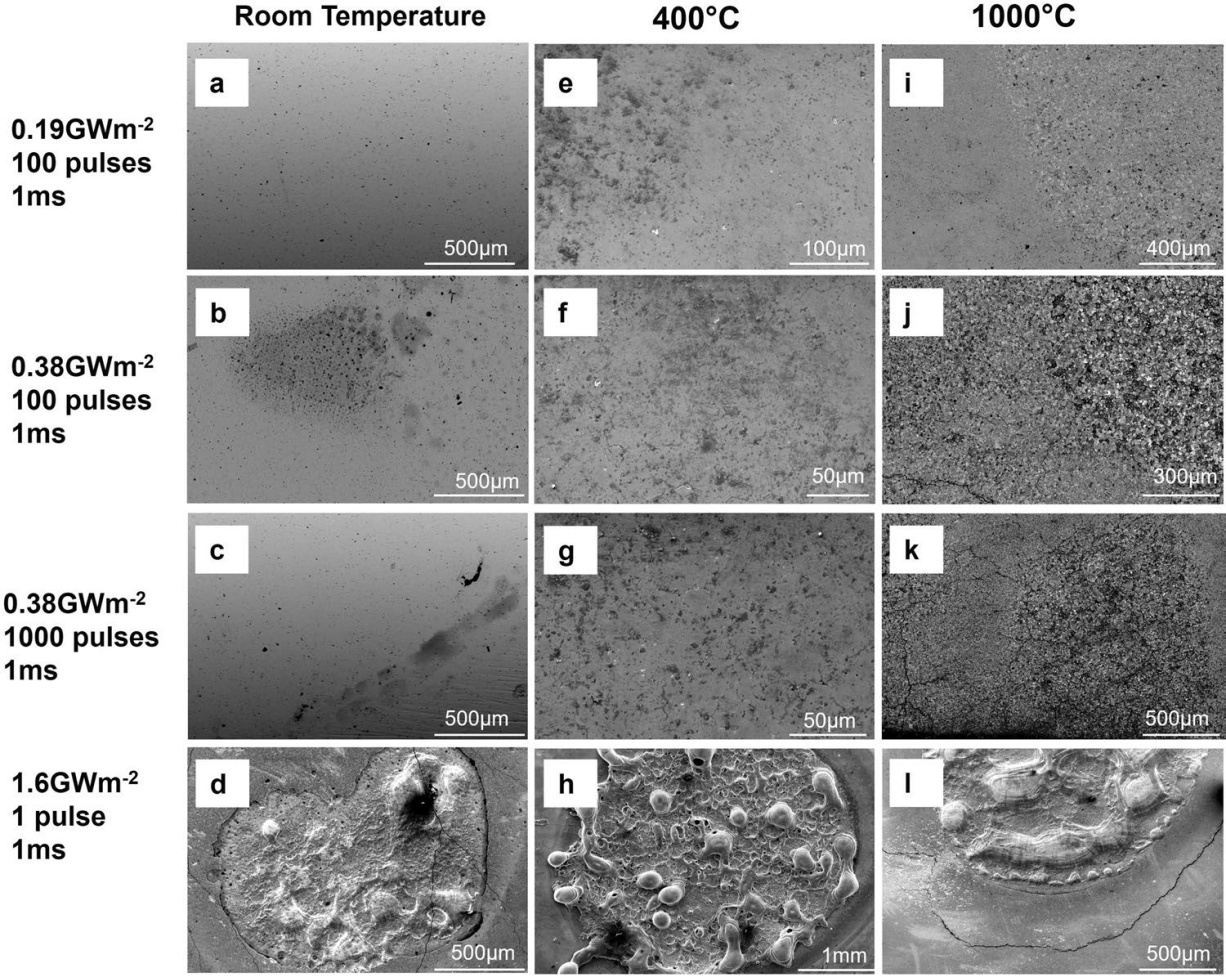
Surface morphology of DSW with 1.1 wt.% ZrC after attacks by high heat flux pulses with different base temperatures. (**a**–**d**) at room temperature; (**e**–**h**) at 400 °C; (**i**–**l**) at 1000 °C. The heat pulse conditions are noted on the left side of each row.

The performance of DSW under high heat flux was tested using the linear plasma device PSI-2 located at Forschungszentrum Jülich, which simulates the transient thermal events with a focused laser beam. To evaluate the performance of DSW under various working conditions, the W-1.1ZrC specimens were held at three different base temperatures (room temperature, 400 °C, and 1000 °C) and three different power densities (0.19, 0.38, and 1.6 GW/m^2^) were deposited on the sample surface. At 0.38 GW/m^2^, the sample was exposed to 100 and then 1000 pulses, in order to investigate thermal fatigue at higher pulse numbers. Postmortem analysis was conducted using SEM. Figure 6 summarizes the surface morphology changes induced by these heat fluxes. During thermal shocks, materials expand but are restrained by colder surroundings, creating compressive stress. If this stress exceeds the material yield strength, it causes plastic deformation. After the thermal shock, the material contracts during cooling but cannot fully revert due to plastic deformation, turning compressive stress into tensile. If the base temperature is below the ductile-to-brittle transition temperature (DBTT), these stresses can result in brittle cracks. Even at temperatures higher than DBTT, multiple thermal shocks could lead to fatigue cracks due to repeatedly applied loads^34^.

As shown in Fig. 6, at room temperature, the W-1.1ZrC samples showed no evidence of crack formation or surface modifications for fluxes of 0.19 or 0.38 GW/m^2^, regardless of the pulse number. At 400 °C, cracks on the order of 10–20 μm started to appear as the pulse energy density increased from 0.19 GW/m^2^ to 0.38 GW/m^2^. At 1000 °C, the samples underwent rapid recrystallization in addition to microscale crack formation even at the lowest power density of 0.19 GW/m^2^. It is worth noting that under the highest power density of 1.6 GW/m^2^, local melting took place regardless of the base temperature, suggesting that the energy density was high enough to raise the local material temperature above the melting point. No apparent fatigue-induced damage events were observed in our experiment since the surface morphologies under 100 and 1000 pulses of 0.38 GW/m^2^ shock are quite similar.

Our results demonstrated that DSW has a superior resistance to cracking than coarse-grained W. A previous study found that for polycrystalline W with grain sizes of 500 μm^2^ or larger, the crack network would form under thermal shocks of 0.19 GW/m^2^ and 1 ms duration at room temperature^34^. In contrast, no obvious change could be found on DSW under a similar thermal shock as described above. Both factors discussed in "Microstructure and chemistry of DSW fabricated by SPS" sect. can contribute to the improved resistance. First, as the dispersoids effectively pin the motion of grain boundaries, DSW maintains a smaller grain size and a higher yield strength, thus becoming more resistant to thermal-induced plastic deformation. More specifically, the average grain size of the W-1.1ZrC in our test was only about 35 μm^2^; Second, since the ductility is improved in DSW^2^, it can tolerate more plastic deformation and result in less cracking.

### Coupling effects of high heat flux and ion fluence on surface erosion

**Figure 7:**
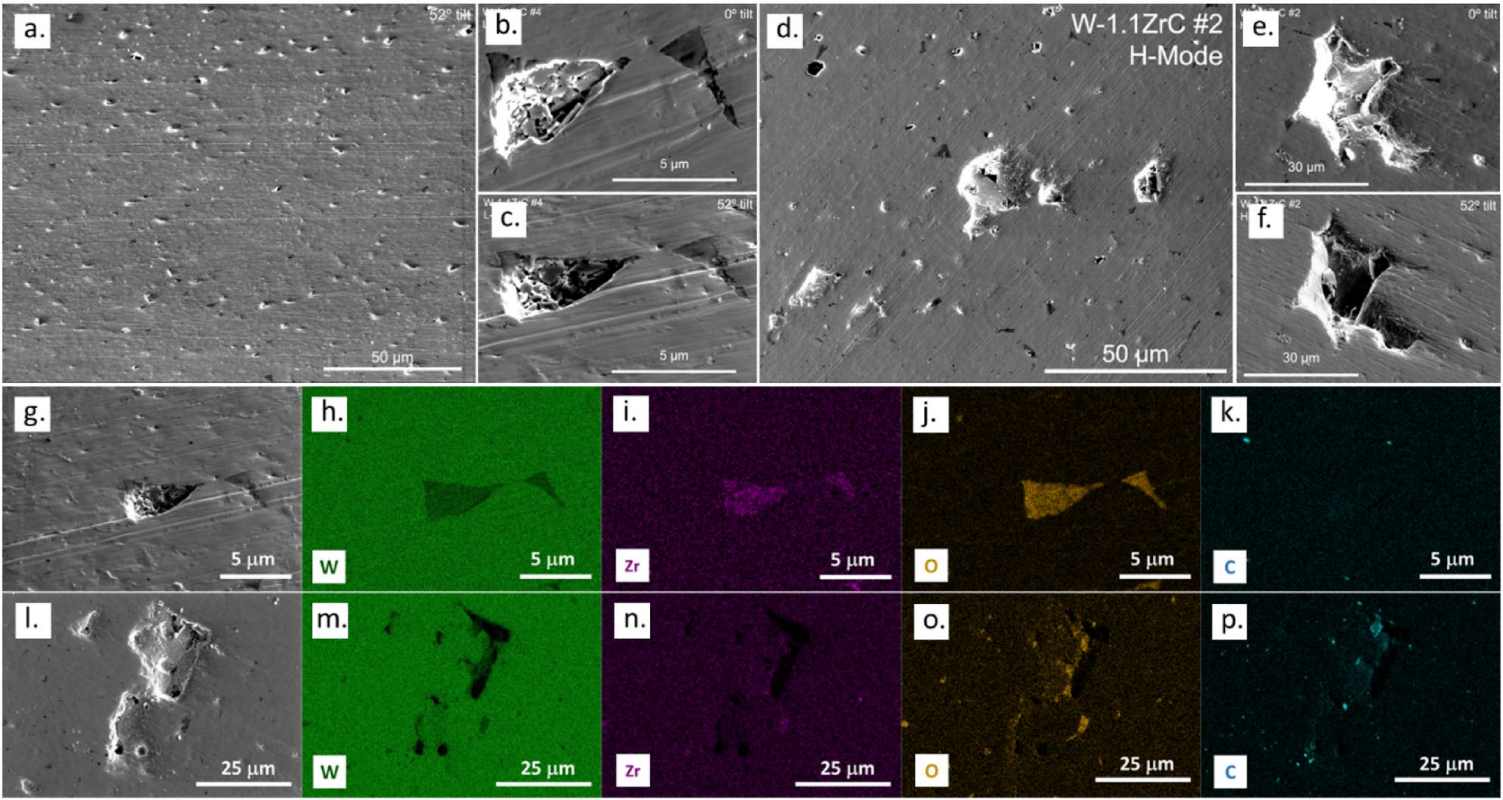
Surface morphology of W-1.1ZrC after exposure in DIII-D. (**a**) Small divots formed under L-Mode; (**b**) Zoomed-in image of a divot at 0° tilt; (**c**) Zoomed-in image of the same divot at 52° tilt, showing the divot is a depression on the surface; (**d**) Both large craters and small divots formed under H-Mode; (**e**) Zoomed-in image of a crater at 0° tilt; (**f**) Zoomed-in image of the same crater at 52° tilt, showing the divot is a depression on the surface; (**g**–**k**) SEM image and corresponding EDS element mapping of a small divot formed under L-Mode; (**l**–**p**) SEM image and corresponding EDS element mapping of a large crater formed under H-Mode.

In order to evaluate the surface stability of DSW in fusion-relevant working conditions, W-1.1ZrC samples underwent exposure to the divertor environment within the DIII-D tokamak. Post-exposure surface analysis was carried out utilizing SEM combined with EDS. As illustrated in Fig. 7a, micrometer-sized divots were present throughout the sample after L-Mode exposure. A closer examination shown in Fig. 7b reveals that these divots align with the dispersoid locations, which have a lower intensity than the W matrix in SEM images. Due to shadowing and electron charging effects, these divots may appear as though they are elevated features. To dispel this illusion, the sample surface was angled to 52° in the SEM, more accurately representing the divots as depressions on the surface (Fig. 7c). EDS element mapping in Fig. 7g–k further demonstrated the spatial correlation between the divots and the dispersoids. Several factors may contribute to the generation of these divots. The dispersoids may experience preferred sputtering, as previous simulation studies revealed that transition metal carbides exhibit a higher sputter yield than the W matrix. Moreover, as discussed in the previous section, oxidation of the ZrC dispersoids could significantly reduce their thermal conductivity, potentially resulting in local hot spots, fractures, and significant mass loss at the dispersoid sites^29,30^.

Besides divots, remarkable surface craters as large as tens of micrometers were observed on the surface of the W-1.1ZrC samples subjected to H-mode plasmas as shown in Fig. 7d. The sample was again tilted in SEM to ascertain these craters as surface depressions (Fig. 7e–f). EDS analysis revealed a higher presence of impurity material in the crater, predominantly C and O, but also traces of B, Na, Si, and metals (Al, Cr, Cu). Given that the plasma-facing materials in DIII-D are primarily graphite and boronization is used to control the plasma impurities, it is plausible that the craters act as plasma-shadowed regions where the impurity elements can be deposited, while the plasma cannot reach the same region to re-sputter the accumulated impurities. These large craters were absent on the L-mode exposed samples. Both sets of samples received similar ion fluences, i.e., 1.62 × 10^23^ m^−2^ and 1.40 × 10^23^ m^−2^ for the H-mode and L-mode exposures, respectively. It indicates that the presence of ELMs and higher heat fluxes play a key role in the generation of these craters. Micrometer-sized craters were observed in previous studies of deuterium-irradiated pure W with controlled pulsed heat fluxes to simulate ELMs. The coupled effects of high deuterium fluence and high heat flux were proposed as the cause of crater formation in these studies. Specifically, deuterium blisters accumulated on the sample surfaces, and the pulsed heat fluxes led to their collapse^35^. It is worth noting that the craters formed on the DSW samples were larger than the craters on pure W in the literature^35^, so future research is needed to fully understand the role of dispersoids in the formation of these large craters.

## Discussion

As evidenced by the two cases examined above, SPS has been proved to be an efficient method to fabricate W-based composites emerging as novel PFMs. We have demonstrated that the unique composite-structure of DSW can substantially enhance the resistance to cracking under high heat fluxes. Our investigations into W-Zr precursors and porous W have revealed that the parameters of SPS markedly impact the microstructures of the resulting composites. This includes factors such as phase fraction, distribution, and grain size of each phase, all of which play crucial roles in determining the performance of the W composites in fusion reactors. Furthermore, specific SPS conditions with higher pressure and temperature loadings led to an extensive formation of intermetallic W_2_Zr phases. This hindered the access of the Zr phase to acids, consequently inhibiting the formation of porous tungsten. These findings underscore the need for systematic studies of SPS parameters to achieve optimal microstructures in the manufactured products. These parameters include, but are not limited to, the sintering temperature, holding pressure, holding time, and powders. Given the vast parameter space, it would be beneficial to develop and apply modeling capabilities for the SPS process, as suggested in some recent studies^36–38^.

As shown in this work and previous research^39,40^, oxygen impurities present a consistent challenge in both types of SPS-fabricated W composites. In the W-Zr precursors, the presence of zirconium oxide in the sample potentially limits the dissolution of Zr placeholders, which is essential for producing porous W. In the DSW composites, the oxidation of carbide dispersoids significantly reduces the material′s thermal conductivity. This could contribute to localized material loss or melting under transient conditions (e.g., ELMs and VDEs) during tokamak operation. Several factors may introduce oxygen impurities into the fabricated W composites. The oxide may originate from the starting powders for sintering, given their high surface area propensity for oxygen absorption^41^. Additionally, oxygen could be introduced from the environment during the sintering process^42^. Even though SPS was performed in a low vacuum condition, the lack of oxygen getters in the system means residual oxygen may still be absorbed onto the powder during the process. Moving forward, it will be important to identify the sources of these impurities and optimize the SPS process to control impurity levels. Promising approaches may involve ball milling the powders in a reducing environment and creating inert environments for the entire sample handling and transfer processes. Simultaneously, gaining a deeper understanding of how oxygen impurities impact the performance of W-based PFMs is essential. This is particularly important considering the challenge of completely eliminating oxygen impurities during the fabrication and usage of W-based composites.

## Summary

In this work, we demonstrated that SPS, including PHSPS, is a versatile and promising method to fabricate W-based composites as PFMs for future high-duty fusion reactors. Specifically, PHSPS provides a promising solution to the large-scale fabrication of porous W with micrometer-sized pores that enables the innovative concept of liquid–metal PFMs, while DSW fabricated using SPS exhibited improved thermomechanical properties over pure W under reactor-relevant conditions. The broad parameter space of SPS offers both opportunities and challenges in optimizing this advanced manufacturing process to achieve optimal microstructures in the fabricated products. Furthermore, we identified that oxygen impurities present a common challenge in both SPS-fabricated systems. Therefore, future research is required to better control these impurities and fully understand their impact on the performance of W-based composites.

## Methods

### SPS fabrication of W-based composite materials

SPS, sometimes referred to as the field-assisted sintering technique (FAST) is a method of fabricating refractory alloys and composites from consolidated powders. The high hardness of W and transition metal carbide (TaC, TiC, ZrC) powders makes them difficult to compact under pressure. In SPS, powders are die-punched via compression at pressures exceeding 50 MPa in a graphite die. The mechanical loading system which consists of die-punched powders also acts as a high-current electrical circuit operating in a controlled atmosphere. Transition metal and carbide powders used in SPS have excellent electrical and thermal conductivities, therefore, ~ 10–20 V loads produce currents on the order of 1 kA and lead to rapid joule heating. With sintering times of 5–10 min, temperatures exceed 1800 °C and pressures exceed 60 MPa for DSW alloys. During sintering, the SPS device maintained a low vacuum (~ 5 Pa). Through varying hold times, pressures, and heating profiles, the thermomechanical properties of DSW alloys are highly tailorable to optimize their performance in a fusion environment. Smaller grain sizes yield desirable properties, including an increase in strength due to a high grain boundary density offering more dislocation barriers. Powder sizes varying from 20 to 800 nm were used in this work for fabricating DSW.

### Fabrication of porous W

**Figure 8:**
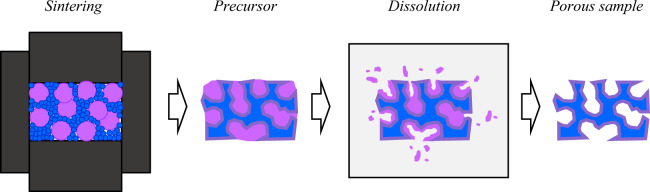
Schematic of PHSPS process. Primary constituent shown in blue, placeholder shown in pink, and potential intermetallic alloy shown in purple.

PHSPS was employed for fabricating porous W that utilizes Zr powder as a place holder, which is dissolved out in acid solutions in post-sintering. The PHSPS method allows for sintering at higher temperatures and pressures than possible with partial sintering and allows for much greater control over the pore parameters. A schematic of the PHSPS process is shown in Fig. 8.

The advantage of PHSPS over a partial sintering method is that it allows for use of higher pressures and temperatures, as the secondary constituent acts as a scaffolding during sintering. This theoretically creates a stronger porous substrate in the end. Additionally, the properties of the porous microstructure (such as porosity, pore size, and ligament size) can be precisely tuned by varying the relative powder sizes and volumetric ratios of the constituents.

Four porous W samples were fabricated using the PHSPS method with Zr placeholders. 99.9% pure W powders of 0.8 and 20 μm, and 99% pure Zr powders of 5 and 75 μm were procured from US Research Nanomaterials. The powders were packed into high-strength graphite dies from California Nanotechnologies and sintered in a Fuji Electronic Industrial Dr. Sinter SPS-725 V sintering press. The sintering parameters used are shown in Table 3. During sintering, the environment was brought down to low vacuum, typically around 5 Pa, and the temperature monitored via an optical pyrometer. The surface of each sintered precursor sample was polished to a near-mirror finish, and then sonicated in acetone for 15 min, before rinsing with DI water. Samples were then placed in acid baths to dissolve out the Zr placeholders and create a porous tungsten surface. They were placed in baths of 1HNO_3_: 9H_2_SO_4_ for up to 25 h. All acids used were concentrated (67% nitric acid, 98% sulfuric acid) ACS grade from Fisher Scientific.

**Table 3:** Sintering parameters for W-Zr samples fabricated via PHSPS.

No.	Powder size (μm)	Vol.% W	Heating rate (°C min^−1^)	Hold time (min)	Hold pressure (MPa)	Hold temperature (°C)
1	W-0.8/Zr-5	60	100	5	50	1700
2	W-20/Zr-75	50	85	5	65	1700
3	W-20/Zr-75	50	85	5	65	1500
4	W-20/Zr-75	40	85	5	50	1400

### XPS characterization of surface chemistry

Surface chemistry of the W composites was assessed using XPS investigations employing a Physical Electronics VersaProbe III instrument equipped with a monochromatic Al K_α_ X-ray source (hν = 1486.6 eV, 200 μm spot size) and a concentric hemispherical analyzer. The emission angle was 45°. Charge neutralization was achieved with a dual-beam charge neutralization system (low-energy electrons and low-energy Ar^+^ ions). Before the experiments, the binding energy scale of the spectrometer was calibrated by setting the Fermi edge of sputtered gold to 0 eV and the Au4f_7/2_ line to 84.0 eV. The acquired spectra were charge referenced by fixing the C1s peak of surface adventitious carbon at 284.8 eV to further correct the charging effect in case of improper electrical contact between the sample and the holder. Measurements were made at a takeoff angle of 45° with respect to the sample surface plane.

High-resolution spectra of the peaks of interest were fitted using a non-linear least-squares method with a mixture of Gaussian and Lorentzian functions by imposing constraints on both the relative intensities of the doublet and spin–orbit splitting while keeping the same full width at half maximum (FWHM) value on similar peaks. Prior to each fit, the background was subtracted using the Shirley method^43^.

### SEM characterization

Mechanical polishing was conducted on samples for SEM analysis. The aim of mechanical polishing is to achieve a surface smooth enough to allow for high-resolution imaging. The first step uses a series of abrasive papers, progressing from coarse to fine grit sizes to reduce surface irregularities. The final step involves using colloidal silica suspensions with a particle size of about 0.05 µm. SEM characterization was conducted using the Apreo field-emission SEM (FESEM) which can achieve nanometer spatial resolution. The Apreo FESEM system also houses integrated detectors for EDS and EBSD, providing a comprehensive analytical suite.

### Testing of DSW under thermal shocks

The linear plasma device PSI-2 located at Forschungszentrum Jülich is used to simulate the damage induced by transient high heat flux events with a focused laser beam. Samples of W-1.1 wt.% ZrC were held at room temperature, 400 °C, and 1000 °C via a temperature-controlled sample holder within the device. Power fluxes of 0.19, 0.38, and 1.6 GW/m^2^ were deposited normal to the sample surface with a spot size of 1 mm^2^. At 0.38 GW/m^2^, the sample was exposed to 100 and then 1000 pulses, in order to investigate thermal fatigue at higher pulse numbers. The samples used in this study have a thickness of 5 mm. According to Eq. (1), the thickness is well above the heat propagation distance for W in the range of power fluxes used for this study^40^. It should be noted that the thermal conductivity of pure W is used to estimate this distance and this value is lower for DS-W due to the presence of oxides and carbides^44^. The actual heat propagation distance for DS-W is lower than the one reported for pure W.

1 $$Heat\,Propagation\;Distance = \left( {\frac{2t\lambda }{{\rho C_{p} }}} \right)^{1/2} = 240\;{\mu m}$$

where $t = 1$ ms is the pulse time, $\lambda = 169\, \frac{{\text{W}}}{{{\text{m}}\;{\text{K}}}}$ is the thermal conductivity, $\rho = 19.3 \;\frac{{\text{g}}}{{{\text{m}}^{3} }}$ is the density of materials, and $C_{p}$ is the heat capacity.

### Testing of DSW in DIII-D tokamak

The DIII-D tokamak is an experimental fusion device and plasma science research facility. Within the facility, the Divertor Materials Evaluation System (DiMES) enables the exposure of samples to the divertor environment in the DIII-D reactor^43^. A total of eight SPS-fabricated DSW samples were exposed using the DiMES system. The DIII-D tokamak has two operational regimes, i.e., the high-confinement mode (H-Mode) and the low-confinement mode (L-Mode). H-Mode features a higher plasma temperature and fusion power density than L-Mode. One DiMES cap holding four DSW samples was exposed to L-mode plasmas and another cap was exposed to H-mode plasmas. Each cap contained four DSW samples: one W-1.1TiC, one W-1.1TaC, and two W-1.1ZrC. The samples exposed to L-Mode plasmas received a particle fluence of 1.40 × 10^23^ m^−2^, and an average heat flux of 0.47 MW m^−2^. Here the particles are mostly deuterium ions with an average energy of 122 eV. The samples exposed to H-mode plasmas received a particle fluence of 1.62 × 10^23^ m^−2^ with the average deuterium ion energy of 182 eV. These H-Mode discharges also had Edge Localized Modes (ELMs), with an ELM period of 10–20 ms. The inter-ELM heat flux was 0.58 MW m^−2^ and the intra-ELM (peak) heat flux was 2.57 MW m^−2^.

### Supplementary Information

Supplementary Information.

## Data Availability

The datasets generated during and/or analyzed during the current study are available from the corresponding author on reasonable request.
