# Author Correction: Discovering tungsten-based composites as plasma facing materials for future high-duty cycle nuclear fusion reactors

**DOI:** 10.1038/s41598-024-69218-5

**Published:** 2024-08-07

**Authors:** Trevor Marchhart, Chase Hargrove, Alexandru Marin, Hanna Schamis, Ashrakat Saefan, Eric Lang, Xing Wang, Jean Paul Allain

**Affiliations:** 1 Ken and Mary Alice Department of Nuclear Engineering, Pennsylvania State University, University Park, PA 16801 USA; 2 Surface Analysis Laboratory, Institute for Nuclear Research Pitesti, 115400 Mioveni, Romania; 3 Department of Nuclear, Plasma and Radiological Engineering, University of Illinois at Urbana-Champaign, Urbana, IL 61801 USA; 4 Department of Nuclear Engineering, University of New Mexico, Albuquerque, NM 87106 USA

Correction to: *Scientific Reports* 10.1038/s41598-024-64614-3, published online 15 June 2024

The original version of the Article contained errors in Figure 6. A wrong scanning electron microscopy (SEM) image was used for panel 6 g, which shows an SEM image acquired near the location of panel 6f with a higher magnification. Additionally, the scale bar for panel 6f is not accurate, and the labels “0.19 GW m^−2^ 1000 pulses 1 ms”, “0.38 GW m^−2^ 1000 pulses 1 ms”, and “0.38 GW m^−2^ 10,000 pulses 1 ms” were incorrect.

**Figure 6:**
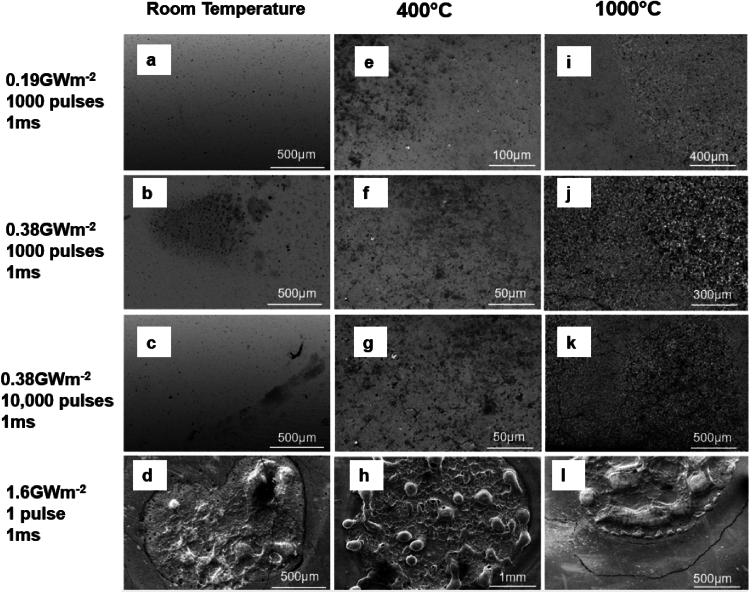
Surface morphology of DSW with 1.1 wt.% ZrC after attacks by high heat flux pulses with different base temperatures. (**a**–**d**) at room temperature; (**e**–**h**) at 400 °C; (**i**–**l**) at 1000 °C. The heat pulse conditions are noted on the left side of each row.

Consequently, in Figure 6,

“0.19 GW m^−2^ 1000 pulses 1 ms”.

now reads

“0.19 GW m^−2^ 100 pulses 1 ms”.

and

“0.38 GW m^−2^ 1000 pulses 1 ms”.

now reads

“0.38 GW m^−2^ 100 pulses 1 ms”.

and

“0.38 GW m^−2^ 10,000 pulses 1 ms”.

now reads

“0.38 GW m^−2^ 1000 pulses 1 ms”.

Additionally, in the Performance evaluation under fusion reactor-relevant conditions section, under the subheading ‘Evolution of surface morphology of DSW under high heat flux’,

“At 0.38 GW/m^2^, the sample was exposed to 1000 and then 10,000 pulses, in order to investigate thermal fatigue at higher pulse numbers.”

now reads

“At 0.38 GW/m^2^, the sample was exposed to 100 and then 1000 pulses, in order to investigate thermal fatigue at higher pulse numbers.”

and

“No apparent fatigue-induced damage events were observed in our experiment since the surface morphologies under 1000 and 10,000 pulses of 0.38 GW/m^2^ shock are quite similar.”

now reads

“No apparent fatigue-induced damage events were observed in our experiment since the surface morphologies under 100 and 1000 pulses of 0.38 GW/m^2^ shock are quite similar.”

The original Figure 6 and accompanying legend appear in this Correction, and the Figure 6g,f were replaced with the correct images.

The original Article has been corrected.

