# Supplementary material for: Discovering tungsten-based composites as plasma facing materials for future high-duty cycle nuclear fusion reactors
Source: Sci Rep. 2024 Jun 15;14:13864. doi: 10.1038/s41598-024-64614-3 (PMC11180118; doi:10.1038/s41598-024-64614-3)
Supplement: Supplementary file 1 — Supplementary Information. [file 41598_2024_64614_MOESM1_ESM.docx]

**Supplementary Materials for**

**Discovering tungsten-based composites as plasma facing materials for future high-duty cycle nuclear fusion reactors**

Trevor Marchhart^1*^, Chase Hargrove^1*^, Alexandru Marin^1,2^, Hanna Schamis^1^, Ashrakat Saefan^1^, Eric Lang^3,4^, Xing Wang^1, #^, and Jean Paul Allain^1, #^

1. Ken and Mary Alice Department of Nuclear Engineering, Pennsylvania State University, University Park, PA 16801, USA

2. Surface Analysis Laboratory, Institute for Nuclear Research Pitesti, 115400 Mioveni, Romania

3. Department of Nuclear, Plasma and Radiological Engineering, University of Illinois at Urbana-Champaign, Urbana IL 61801, USA

4. Department of Nuclear Engineering, University of New Mexico, Albuquerque, NM 87106, USA

* Authors contributing equally to this work

# Corresponding authors: [xvw5285@psu.edu](mailto:xvw5285@psu.edu) (Xing Wang), [allain@psu.edu](mailto:allain@psu.edu) (Jean Paul Allain)

**1.Three-point bending tests of W-Zr samples**

In order to get an initial understanding of the mechanical properties of the W-Zr composite with porous tungsten on the surface, miniature three-point bending tests were performed on Sample 1 and Sample 4 as defined in the main text. Each precursor sample was cut into 16×3×~0.6 mm rectangular prisms via wire electrical discharge machining (EDM), then subsequently exposed to the acid bath before testing. Scanning electron microscopy (SEM) imaging in Fig. S1 showed that only the outer ~150 μm surfaces of the specimen became porous. Due to the small size of the samples produced via SPS, a full-size standardized test method was not able to be used for three-point bending. Rather, a custom miniature three-point bend loading structure was developed utilizing 0.5 mm tungsten carbide rods as loading pins, with a span of 15mm. The span/thickness ratio of 15/0.6 was deemed acceptable to prevent significant shear effects. This custom fixture was used with an MTS Criterion 100 kN load frame with a high resolution 50 N load cell. Error for each flexural modulus and strength calculated was determined assuming a conservative ±0.1 N error on the load cell force measurement and a ±0.02 mm error on the thickness measurement with calipers. Two prisms of Sample 1 and three prisms of Sample 4 were tested. Table S1 displays the measured thickness, flexural modulus, and flexural strength of each tested prism.


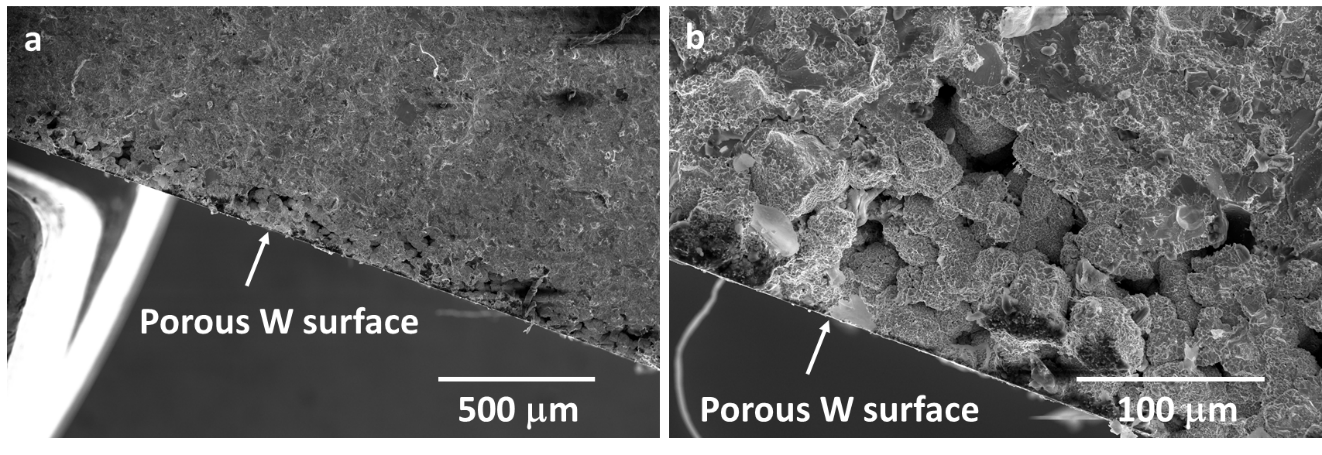


**Figure S1**. SEM cross-sectional view of a W-Zr precursor after acid treatment. (a) Lower magnification image revealing porous W formation near the surface; (b) Higher magnification image of the same sample detailing the cross-section.

Since the outer ~150 µm of each specimen became porous, the measured mechanical properties must be taken as qualitative, as the porous parts of the specimen will have significant effects on the overall strength at this scale. These results in Table S1 suggest a correlation between sintering temperature and strength, likely due to further densification of the precursor at higher temperatures [1]. Additionally, Sample 1 contains 60 vol. % W compared to 40 vol. % for Sample 4 (refer to Table M1 in the main text). The higher W concentration strengthens the dense regions of Sample 1 because of tungsten's higher modulus of elasticity, approximately 400 GPa, compared to that of zirconium, which is around 95 GPa. Besides, the higher W concentration in Sample 1 also strengthens the porous regions in Sample 1, as it is theoretically 60% dense as opposed to 40% dense in Sample 4. Assuming elastic and flexural moduli are comparable for a given material, the reported values in Table S1 are comparably low to that of both W and Zr. However, if only the dense portions of each sample are considered (i.e., thicknesses of 0.3-0.4 mm, which is obtained by subtracting the thickness of porous W on both sides of the prism), the moduli are brought up to ~150-300 GPa – closer to what the expected value might be for a W-Zr alloy of this nature. Additionally, the porous surfaces on either side of the rectangular prism are likely acting as crack initiation points, further decreasing the flexural strengths.

**Table S1.** Flexural moduli and strengths of W-Zr specimen tested under three-point bending

|  | **Sample 1 Prism a** | **Sample 1**  **Prism b** | **Sample 4**  **Prism a** | **Sample 4**  **Prism b** | **Sample 4c**  **Prism c** |
| --- | --- | --- | --- | --- | --- |
| Thickness (mm) | 0.63 | 0.63 | 0.59 | 0.57 | 0.58 |
| Flexural modulus (GPa) | 70.1 ± 6.7 | 75.5 ± 7.2 | 41.5 ± 4.3 | 59.0 ± 6.3 | 47.6 ± 5.0 |
| Flexural strength (MPa) | 274.0 ± 17.5 | 260.5 ± 16.6 | 216.1 ± 14.8 | 252.0 ± 17.8 | 206.8 ± 14.4 |
